# The Edinburgh Medical and Surgical Journal

**Published:** 1834-01-01

**Authors:** 


*The Edinburgh Medical and Surgical Journal*, No. CXVII. October, 1833.

OUR excellent contemporary is so rich in original communications, that it has all the interest of a volume of good medical essays, which it would be unpardonable to pass over in silence; and we shall therefore, from time to time, gratify our readers with a short abstract of the more prominent points contained in it, occasionally indulging in a word or two of comment, approval, or dissent.

Mr. SANDWITH has contributed some remarks on *Scarlatina*, with an account of the disease as it appeared epidemically at Bridlington, in 1831. He insists very strongly on the advantage to be derived from bleeding.

Mr. WISHART has a case of *Extirpation of the Eyeball*, in which the patient, a girl of thirteen, did well. The eye itself was sound, but was pushed out of its orbit by an adhering tumour.

There is a very interesting paper by Dr. OGSTON, entitled *Phenomena of the more advanced Stages of Intoxication, with Cases and Dissections.* The materials were chiefly derived from cases treated at the police-office in Edinburgh, and the following is an abstract of some of their principal points:

" *Tabular View of the Phenomena of the more advanced Stages of Intoxication.*

| Case. | Age. | Sex. | States of the Pupil. | Pulse. | Sensorium. | Extrem. | Face. | Breathing. | Termination. |
|---|---|---|---|---|---|---|---|---|---|
| 1 | 18 | Male. | Dilated. | Impercept. | Prof. coma. | Cold. | Sl. flushed. | Slow. | Stupor, coldness. |
| 2 | 40 | do. | Str. do. | do. | do. do. | do. | Pale. | Laborious. | Fatal in 3-4 hour. |
| 3 | 60 | Fem. | do. | do. | do. | do. | Flushed. | Slow. | Rigors, immediate recovery. |
| 4 | 40 | do. | do. | do. | Prof. do. | do. | Pale. | do. | Immediate recov. |
| 5 | 28 | do. | Str. do. | do. | do. do. | Very do. | Swol.livid. | Laborious. | Fatal. |
| 6 | 30 | Male. | do. do. | do. | do. | do. | Pale. | Slow. | Return of pulse & sensation, pupil contracting. |
| 7 | 60 | Fem. | do. | do. | Prd. do. | Very do. | Flushed. | do. | Stupor, coldness. |
| 8 | 22 | Male. | Str. do. | do. | do. do. | do. do. | Pale. | Laborious. | Fatal. |
| 9 | 38 | Fem. | do. do. | do. | do. do. | do. | Sl. livid. | Slow. | Coldness, rigors. |
| 10 | 30 | Male. | do. do. | Feeb.slow. | do. | do. | Natural. | Laborious. | Delirium, succeed. by stupor. |
| 11 | 35 | do. | do. do. | 68, soft. | do. | Nat.warm. | Very pale. | Slow. | Coma for 8 ho. |
| 12 | 50 | do. | do. | Soft, freq. | do. | do. do. | Flushed. | Rapid. | Convulsions. |
| 13 | 36 | do. | Mod. do. | Feeble. | do. | Cold. | Pale. | Slow. | Stupor. |
| 14 | 27 | Fem. | Str. do. | Full, slow, feeble. | Prd. do. | Very do. | Flushed. | Very do. | Convulsions, hys. and stupor. |
| 15 | 15 | Male. | do. | Do. soft. | do. | Coldish. | do. | Calm. | Noisy delirium, ending in stupor. |
| 16 | 13 | do. | Str. do. | Feeble. | Stupor. | Cold. | do. | Slow. | Immediate recov. |
| 17 | 19 | Fem. | do. do. | Full, slow. | Coma. | do. | do. | do. | Noisy delirium. |
| 18 | 30 | do. | do. | 72, feeble. | Prd. do. | Coldish. | Pale. | do. | Stupor, nausea. |
| 19 | 25 | do. | Str. do. | Soft, full. | do. do. | Nat. warm | do. | Calm. | Immediate recov. |
| 20 | 27 | do. | do. | 84, feeble. | do. | Cold. | Flushed. | Slow. | Stupor, pulse rising to 104. |
| 21 | 28 | Male. | Contrac. | Full. | Stupor. | Nat. warm. | Pale. | Stertorous. | Stupor. |
| 22 | 70 | do. | Much do. | Slow. | Prd. coma. | do. do. | do. | do. | Coma and stertor for 8 hours. |
| 23 | 28 | Fem. | do. do. | Do. indist. | do. do. | Cold. | do. | do. | Do. do. for 16 h. |
| 24 | 32 | Male. | do. | 108, soft. | do. do. | Nat. warm. | Pale and flush.alter. | Do. and puffing. | Symptoms cont. 18 hours, fatal. |
| 25 | 19 | Fem. | Much do. | 79, rather firm. | do. do. | Warm. | Pale. | Stertorous. | Coma for 6 ho. |
| 26 | 27 | do. | do. do. | 84, feeble. | do. | Nat. warm. | do. | do. | Stupor for 6 ho. pulse 100, pupil dilated. |

Bleeding is injurious. One of the best internal remedies is ammonia, but, if there is unmanageable delirium, tartar emetic should be given in nauseating doses. Cold affusion is to be used "where the temperature of the head is steadily high, and that of the surface generally not too much reduced."

Dr. MAUNSELL has given *a Report of the Obstetric Practice of the Wellesley Female Institution.* In the year 1832 the number of cases attended was 442. "Of these, nineteen were abortions, leaving a balance of 423 actual labours. From these were produced 431 children, there having been eight cases of twins, or one in $52\frac{7}{8}$." It is remarkable that, of these, the number of females was 220, and of males 211; and a still more remarkable statement is given in a note, on the authority of Dr. Rutty, by which it would appear that from 1757 to 1770, the female births in Dublin were considerably more numerous than the male ones. We suspect some error in this. Dr. Merriman informs us that 97,057 were delivered in the Dublin Lying-in Hospital, from 1757 to 1820, and that about twelve males were born to eleven females. (*A Synopsis, &c.*, 4th edit., p. 342.)

Among the Wellesley Institution cases there were four arm-presentations, in two of which spontaneous evolution took place.

Dr. Maunsell says that, "among forty-eight healthy women, taken indiscriminately, mostly in the eighth or ninth month of pregnancy, the pulse was, in thirty-two of them, above 100, in many 120, and in one 144." Now it is commonly stated, by writers on obstetric auscultation, that the pulsations of the fœtal heart are about twice as numerous as those of the mother's. If therefore it were common for a pregnant woman to have a pulse of 120, the fœtal pulsations would be too numerous to be counted. Dr. Maunsell's objections, of course, depend on this supposition, which, we will confess, is quite contrary to the result of our experience.

Dr. Dymock, while pursuing his anatomical studies in the year 1830, met with a case of *supernumerary cervical ribs.* He refers to several instances of the same anomaly recorded in the annals of medicine, but does not quote Soemmering, who says, "*Hunauld, Memoir. de l'Acad. Royale des Sciences,* 1741, parum similem costam supernumerariam costæ primæ veræ, *Leveling Obs. Anat. Rariores, Ingolst.* 1786, magis similem describunt, in mea collectione costa supernumeraria costæ primæ veræ omnium maxime similis asservatur, attamen costa supernumeraria ad manus est, minor, quam in Hunauld observatione, costæ primæ similis, ultima enim colli vertebra multum longa, contracta, divisa sistitur." (*S. Th. Soemmering, de Corporis Humani Fabricâ,* tom. i., p. 250.) Dr. Dymock mentions Hunauld's case, but says it appeared in the Memoirs for 1740.

Mr. Syme, in his Clinical Report, gives two additional cases, in which excision of the elbow-joint was successfully performed.

Dr. Coldstream has furnished a good account of the *Topography and Climate of Torquay, in Devonshire.* Those who know what delicate thermometers plants are, will require no other commendation of the mildness of a Torquay winter than a list of the plants which it fosters.

"The following plants are considered, in the gardens around

Torquay as hardy exotics, and usually are allowed to remain in the open border during winter:

| | |
|---|---|
| Azalea Indica, | Magnolia conspicua, |
| Aster Capensis, | Mespilus Japonica, |
| Agave Americana, | Myrtus communis, |
| Bignonia Pandoræ, | Laurus camphora, |
| Cactus speciosus, | Metrosideros floribunda, |
| Calceolaria plantaginea, | Pœonia arborea, |
| ——— corymbosa, | Punica granatum, |
| Cassia Capensis, | Salvia purpurea, |
| Citrus medicus, | Verbena malendris, |
| Coronilla glauca, | Yucca aloifolia, |
| Dracocephalum Canariense, | —— gloriosa." |
| Fuchsia coccinea, | (P. 353.) |

Invalids labouring under chronic bronchitis, or rheumatism, are especially benefited by the climate of this place.

Mr. KEITH, of Aberdeen, gives an account of two cases in which he successfully performed the operation of Lithotrity. The first patient, aged seventy-two, was suffering from the presence of a calculus an inch and a half in diameter. The first sitting took place April 23d, 1833.

"The catheter being withdrawn, the percussor was introduced, and a stone of the dimensions stated readily seized. The instrument secured in the fixed point, or moveable vice of Baron Heurteloup's bed, on which the patient reclined, the hammer was applied, and the stone shattered to pieces. A large fragment was again seized and crushed. A third seizure was effected, but a fold of the mucous membrane of the bladder was felt between the teeth of the percussor and the fragment; and the latter was accordingly dropped.

"The time prescribed (fifteen minutes) having expired, nothing farther was attempted. In a few minutes Mr. F. made water freely, and discharged a quantity of small sand and gravel, composed of lithic acid; and several fragments followed in the course of the night. The urine was tinged with blood, but no hemorrhage whatever followed. His only complaint was of slight heat and scalding along the urethra. He passed a tolerable night, and was out next morning before seven o'clock at his usual walk." (P. 480.)

After several other sittings at various intervals, on the 20th of July the bladder was found to be clear. The collected fragments of the stone weighed an ounce. Among others who witnessed these operations was Mr. Liston, of Edinburgh.

In the second case the symptoms were much more violent, and the patient (sixty years of age,) was evidently suffering from cystitis. He applied to Mr. Keith on the 5th of May,

1833, but the first sitting did not take place till July the 30th, after the inflammation of the bladder had been subdued, or nearly so, by antiphlogistic treatment.

"Having placed him on the rectangular bed, the bladder was injected with warm water to seven ounces, and the percussor introduced. The stone was readily seized and crushed, as were three large fragments subsequently. The bladder was afterwards washed out by a few ounces of water, and the operation finished in fourteen minutes. A tea-spoonful of fragments came away, and a quantity of detritus. He complained only of slight scalding along the urethra, and a feeling as if the neck of the bladder had been rather forcibly dilated that night. Next day the stone symptoms had almost departed; and on the night of August the 1st, he slept from eight P. M. to eight next morning, which he had not done for years. Fragments came away for two or three days.

"On the 3d of August, as the bladder was quiet, and all irritation arising from the former operation gone, he was again operated on, and four fragments crushed. On the 13th, five fragments were comminuted; on the 22d, seven fragments seized and broken, the operation in whole occupying six minutes.

"Injecting the bladder being now rendered unnecessary, from his being able with comfort to retain his urine for five hours prior to the operation, on the 28th, seven small fragments were destroyed. Each of these operations was followed by the discharge of a quantity of debris. On the 3d of this month, September, the bladder was carefully explored, and two fragments, measuring a quarter of an inch in diameter, discovered, picked up, and destroyed; the sand came away, and the bladder was clear. Yesterday morning, at six A. M. he went to his work, perfectly well." (P. 482.)

Mr. Keith disapproves of the anodyne clyster, on account of the parching thirst which it occasions.

Mr. Liston has also performed this operation in two cases, and with perfect success. He says:

"Notwithstanding my disapproval of "the hammering of stones in the bladder," expressed in my Elements of Surgery, I have deemed it incumbent on me to give the practice a fair trial, the improved instruments appearing both simple and efficient.

"I have, accordingly, employed the *percuteur* in two cases. The result has proved so far beyond my expectation, that I send you the following short statement for publication, hoping that it may induce others to make trial of the apparatus. For it is only from the accumulated experience of the regular surgeons, that we can hope for certain advancement in lithotrity.

"The first patient on whom I employed the percuteur thus describes his case.

"*Edinburgh; 9th September,* 1833.

"Sir: After being afflicted for upwards of two years with what I supposed a complaint in my bladder, I waited upon you for advice.

Upon sounding, you discovered there were several stones in my bladder, and immediately extracted (by Weiss's forceps) one much larger than I conceived possible to come through the urethra. At my second and third visits, eight or ten smaller stones were extracted; and by using sweet spirits of nitre, and drinking largely of barley-water, as you advised, I passed, in the course of a few weeks, from sixty to seventy pieces of stone, varying from the size of a small white pea, to that of a small horse-bean.

"At my fourth and fifth visits, you ground down with an instrument, which I suppose of French invention, (Civiale's improved three-branched instrument,) a stone supposed nearly an inch in span, and another about the size of a Spanish nut. By again using spirit of nitre, and drinking barley-water, many small fragments were passed.

"After a lapse of several months, I again waited upon you, when you ground down with a new instrument (the percuteur) a small stone; and, in fourteen days thereafter, when I again waited upon you, you ground down two stones, one of them of considerable size. Agreeably to your instructions, on both these occasions I used sweet spirit of nitre, and drank a good deal of barley-water, which caused a great number of fragments of stone to come away, and stuff like slaked lime.

"The pain and uneasiness caused by the new instrument was infinitely less than what was caused by the other.

"When I waited upon you, on Sunday the 26th ult., fourteen days after the last operation, you sounded me two or three times, without discovering any thing in the bladder. You then injected some warm water, and sounded again, with the same result.

"Since the last operation with the new instrument, I have been entirely free from pain, and now make water with as much freedom and ease as ever I did in my life. Thus, you have cured me of a most distressing complaint, for which I can never be sufficiently thankful and grateful to you. I am, sir, &c.

"J. M."

"The working of Civiale's apparatus in this case produced considerable pain and irritation, whereas the percuteur caused almost no pain whatever, the patient merely complaining of the annoying sensation which accompanies a strong desire to make water. There was also no admixture of blood with the urine that escaped during the operation, showing that no injury had been done to the coats of the bladder." (P. 483.)

In the second case, in which the patient was a labourer, aged fifty-four, Mr. Liston again employed the *percuteur*. At the date of his communication there had been five sittings, and Mr. Liston says, "perhaps one other sitting, certainly not more, may yet be necessary."

In concluding this notice, we cannot refrain from again expressing our admiration of this well-conducted Journal, which is not merely the herald of medical discoveries, but contributes to the progress which it records.

